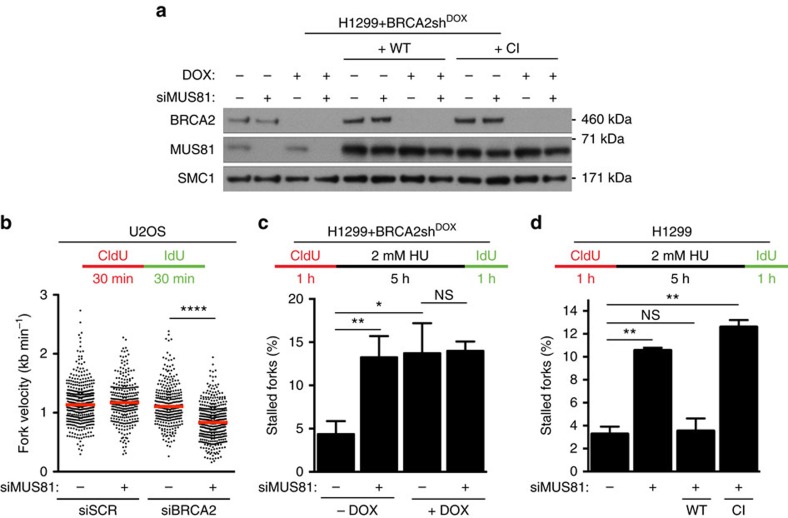# Corrigendum: MUS81 nuclease activity is essential for replication stress tolerance and chromosome segregation in BRCA2-deficient cells

**DOI:** 10.1038/ncomms16171

**Published:** 2017-10-26

**Authors:** Xianning Lai, Ronan Broderick, Valérie Bergoglio, Jutta Zimmer, Sophie Badie, Wojciech Niedzwiedz, Jean-Sébastien Hoffmann, Madalena Tarsounas

Nature Communications 8: Article number: 15983; DOI: 10.1038/ncomms15983 (2017); Published: 07 17 2017; Updated: 10 26 2017

In this Article, there are errors in the labelling of the *y* axis in Fig. 1a and Supplementary Fig. 1b. The labels ‘20’, ‘40’, ‘60’, ‘80’ and ‘100’ should have been ‘0.5’, ‘1.0’ and ‘1.5’ in Fig. 1a and the labels ‘50’, ‘100’, ‘150’ and ‘200’ should have been ‘1’, ‘2’ and ‘3’ in Supplementary Fig. 1b. The correct versions of these figures appear below as Figs 1 and Figs 2, respectively.

## Figures and Tables

**Figure 1:**
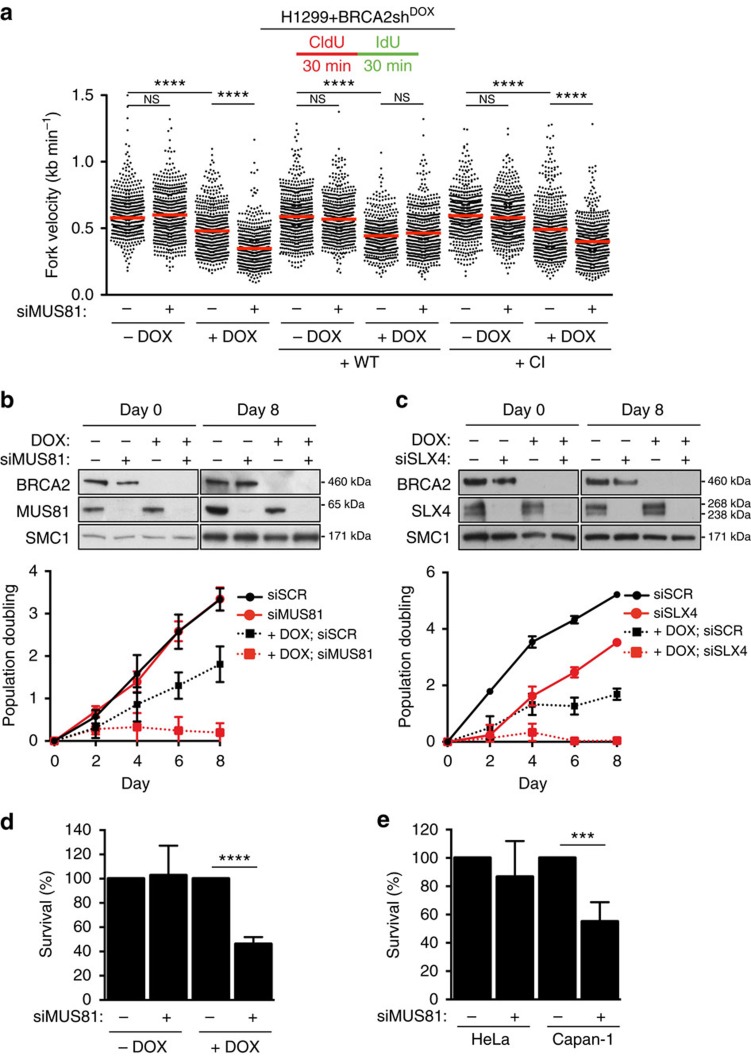


**Figure 2:**