# Supplementary material for: MUS81 nuclease activity is essential for replication stress tolerance and chromosome segregation in BRCA2-deficient cells
Source: Nat Commun. 2017 Jul 17;8:15983. doi: 10.1038/ncomms15983 (PMC5520020; doi:10.1038/ncomms15983)
Supplement: Supplementary Information [file ncomms15983-s1.pdf]

File name: Supplementary Information
Description: Supplementary figures and supplementary tables.

File name: Peer review file
Description:

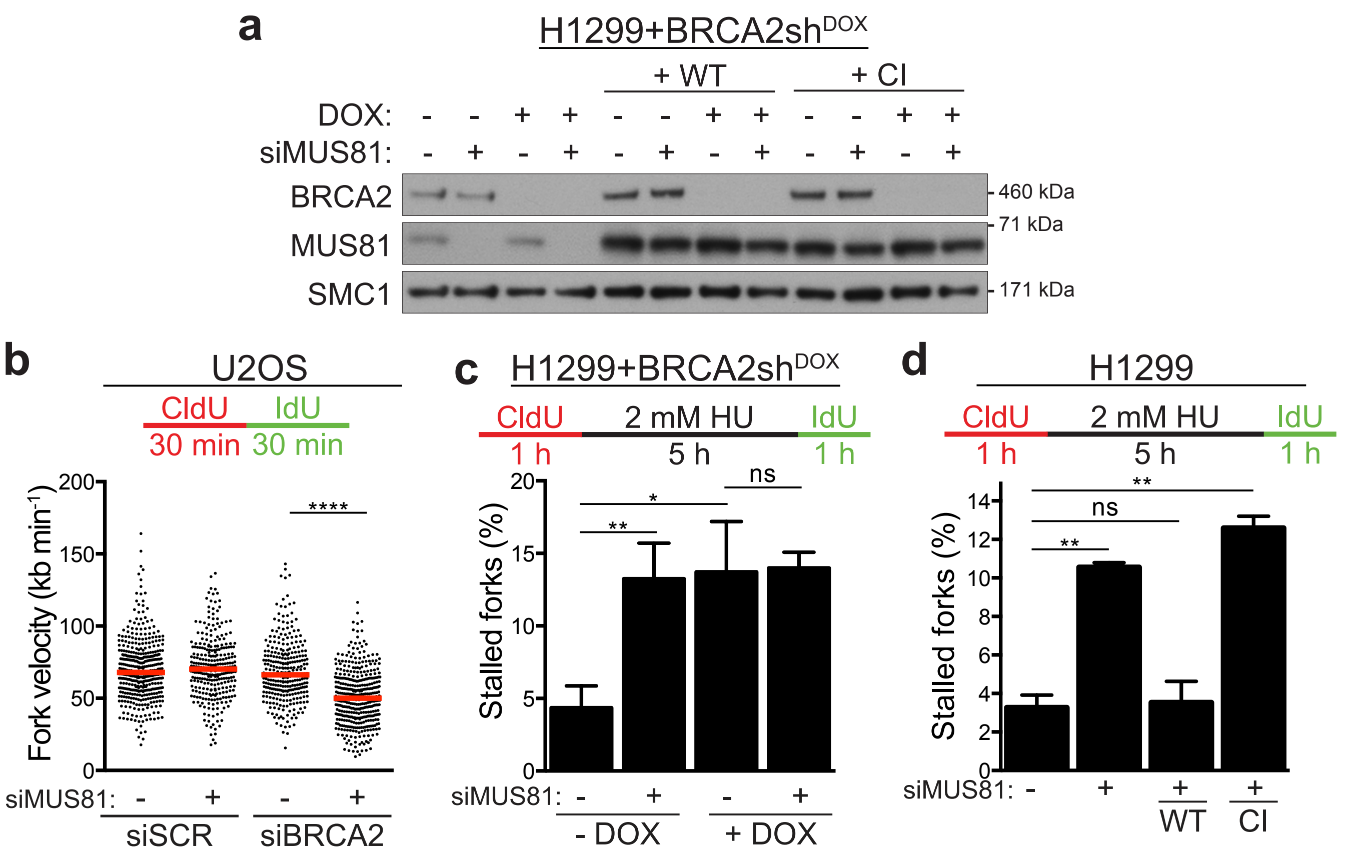

Supplementary Figure 1. MUS81 is required for DNA synthesis in human BRCA2-deficient cells. (a) H1299 cells carrying a doxycycline (DOX)-inducible BRCA2 shRNA were transfected with control or MUS81 siRNAs and immunoblotted as indicated. SMC1 was used as a loading control. Stable cells lines expressing either wild type (WT) or catalytically inactive (CI) human MUS81 were similarly processed. (b) U2OS cells were transfected with control, BRCA2 and/or MUS81 siRNAs and processed for DNA fiber analysis 72 h later, as outlined in the inset, followed by quantification of CldU+IdU track length. Fork velocity was calculated using a conversion factor of 1 μm = 2.59 kb min^{-1}. Red bars indicate mean (n=3). ****, $p < 0.0001$ (two-tailed Mann-Whitney test). (c) H1299 cells carrying a DOX-inducible BRCA2 shRNA were transfected with control or MUS81 siRNAs and processed for DNA fiber analysis after 24 h as outlined in the inset, followed by quantification of the frequency of stalled forks (tracks containing CldU label only). Error bars indicate SD (n=3). *, $p < 0.05$; **, $p < 0.01$ (unpaired two-tailed t test). HU, hydroxyurea. (d) Stable H1299 cells expressing either WT or CI MUS81 were treated as in (c) followed by quantification of the frequency of stalled forks. Error bars indicate SD (n=2). **, $p < 0.01$ (unpaired two-tailed t test).

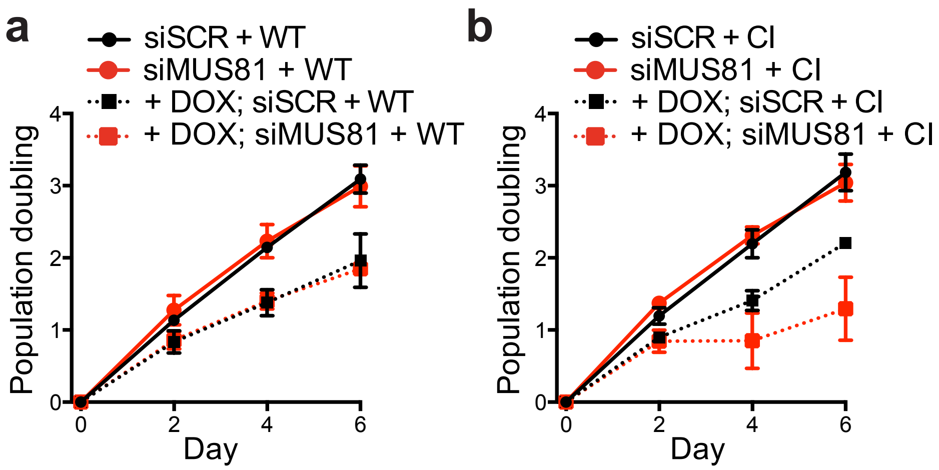

Supplementary Figure 2. MUS81 catalytic activity is required for the survival of BRCA2-deficient cells. Stable H1299 cell lines carrying a DOX-inducible BRCA2 shRNA and expressing either WT (a) or CI (b) human MUS81 were transfected with control or MUS81 siRNAs and processed for proliferation assays after 24 h. siRNAs were re-transfected at an interval of four days. The proliferation graphs shown are representative of three independent experiments. Error bars represent SD of triplicate values obtained from a single experiment.

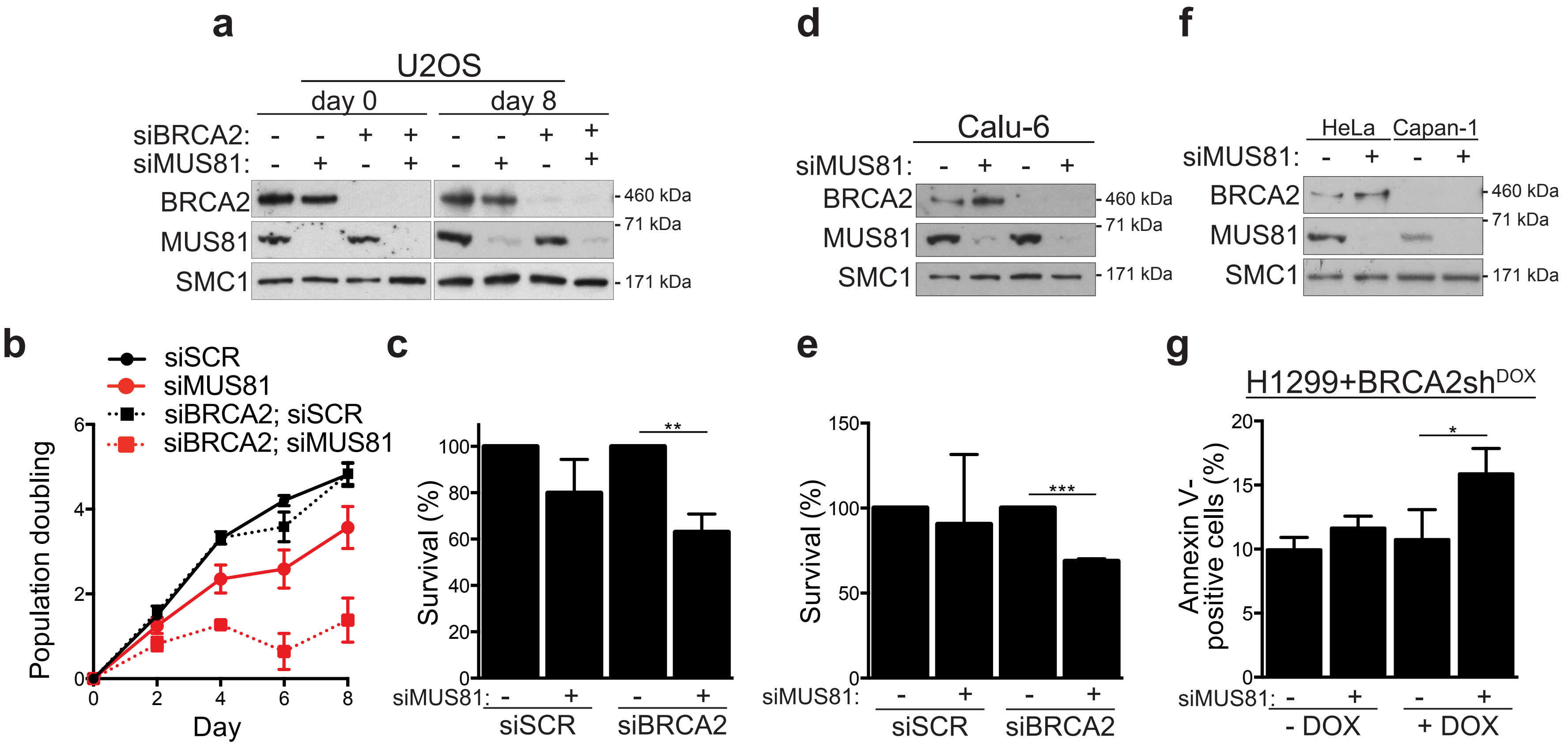

Supplementary Figure 3. MUS81 inactivation decreases survival of BRCA2-deficient cells. (a) U2OS cells were transfected with control, BRCA2 and/or MUS81 siRNAs and processed for proliferation assays after 24 h. siRNAs were re-transfected at an interval of four days. Cell extracts prepared at indicated timepoints were immunoblotted as indicated. SMC1 was used as a loading control. (b) Representative proliferation graph of three independent experiments of cells treated as in (a). Error bars represent SD of triplicate values obtained from a single experiment. (c) Cells treated as in (a) were processed for clonogenic survival assays. Colonies were stained after 10-14 days. A survival graph representative of two independent experiments is shown. Error bars represent SD of triplicate values obtained from a single experiment. **, $p < 0.01$ (unpaired two-tailed *t* test). (d) Calu-6 cells were transfected with control or MUS81 siRNAs and immunoblotted as indicated. SMC1 was used as a loading control. (e) Cells treated as in (d) were plated 24 h later for clonogenic survival assays. Colonies were stained after 10-14 days. Error bars represent SD (n=2). ***, $p < 0.001$ (unpaired two-tailed *t* test). (f) HeLa or Capan-1 cells were transfected with control or MUS81 siRNAs and immunoblotted as indicated. SMC1 was used as a loading control. (g) H1299 cells expressing DOX-inducible BRCA2 shRNA were transfected with control or MUS81 siRNAs and processed 72 h later for Annexin V staining. Error bars represent SD (n=3). *, $p < 0.05$ (unpaired two-tailed *t* test).

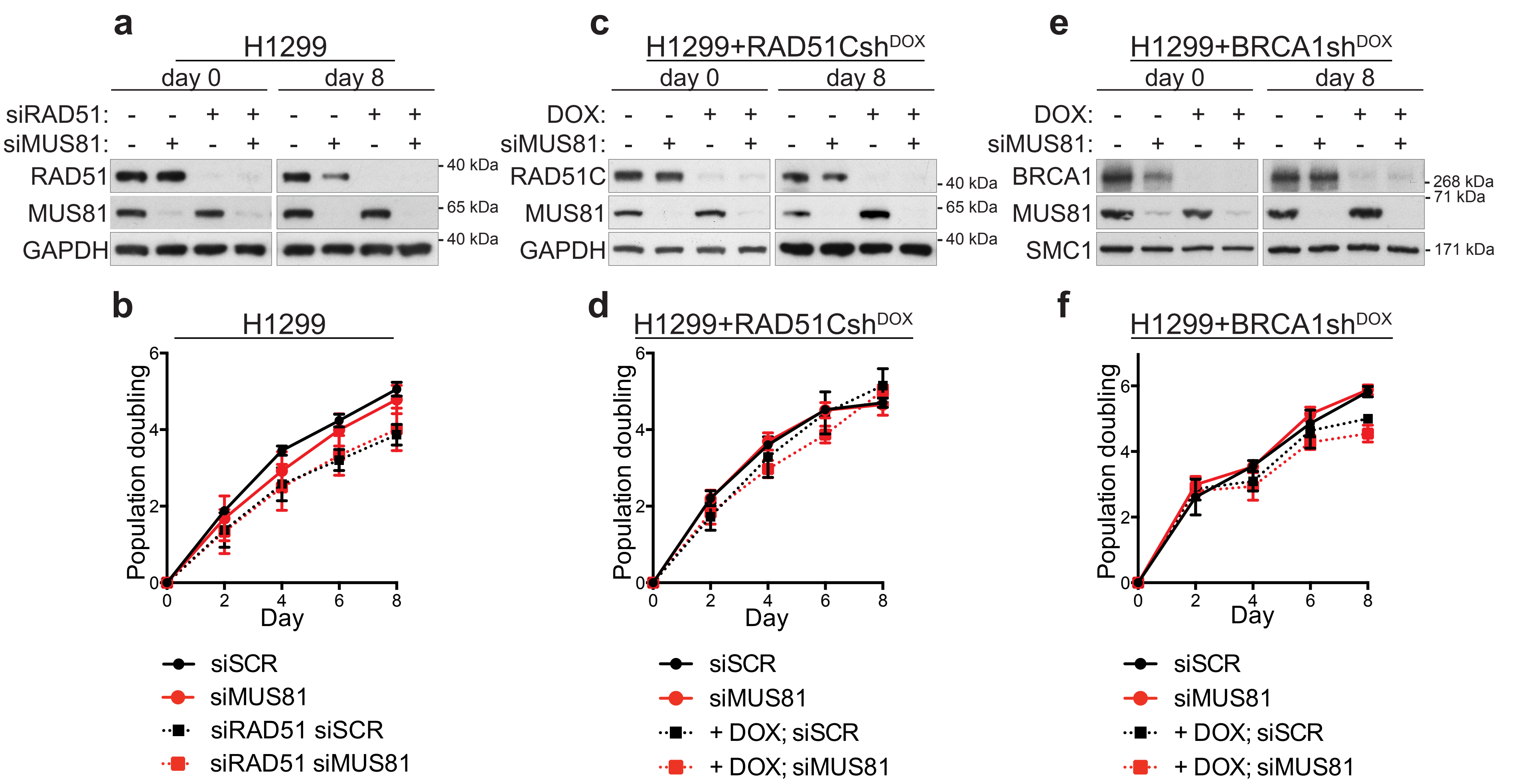

Supplementary Figure 4. MUS81 depletion does not affect proliferation of RAD51-, RAD51C- or BRCA1-deficient human H1299 cells. (a) H1299 cells were transfected with control, RAD51 esiRNA and/or MUS81 siRNAs and processed for proliferation assays after 24 h. siRNAs were re-transfected at an interval of four days. Cell extracts prepared from indicated time points were immunoblotted as shown. GAPDH was used as a loading control. (b) Representative proliferation graph of three independent experiments of cells treated as in (a). (c) H1299 cells expressing a DOX-inducible RAD51C shRNA were transfected with control or MUS81 siRNAs and processed as in (a). (d) Representative proliferation graph of two independent experiments of cells treated as in (c). (e) H1299 cells expressing DOX-inducible BRCA1 shRNA were transfected with control or MUS81 siRNAs and processed as in (a). SMC1 was used as a loading control. (f) Representative proliferation graph of two independent experiments of cells treated as in (e). All error bars represent SD of triplicate values obtained from a single experiment.

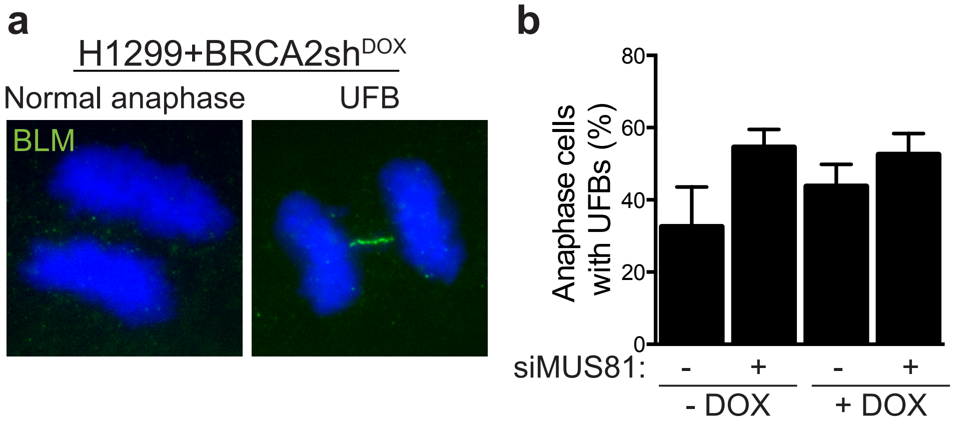

Supplementary Figure 5. UFB frequency in H1299 cells lacking MUS81 and/or BRCA2. (a) H1299 cells expressing DOX-inducible BRCA2 shRNA were transfected with control or MUS81 siRNAs and mitotic cells were immunostained for BLM (green) after 72 h. DNA was counterstained with DAPI (blue). Representative immunofluorescence images of anaphase cells analysed for ultra-fine bridges (UFBs) are shown. (b) Quantification of anaphase cells with UFBs treated as in (a). All error bars indicate SD (*n*=4).

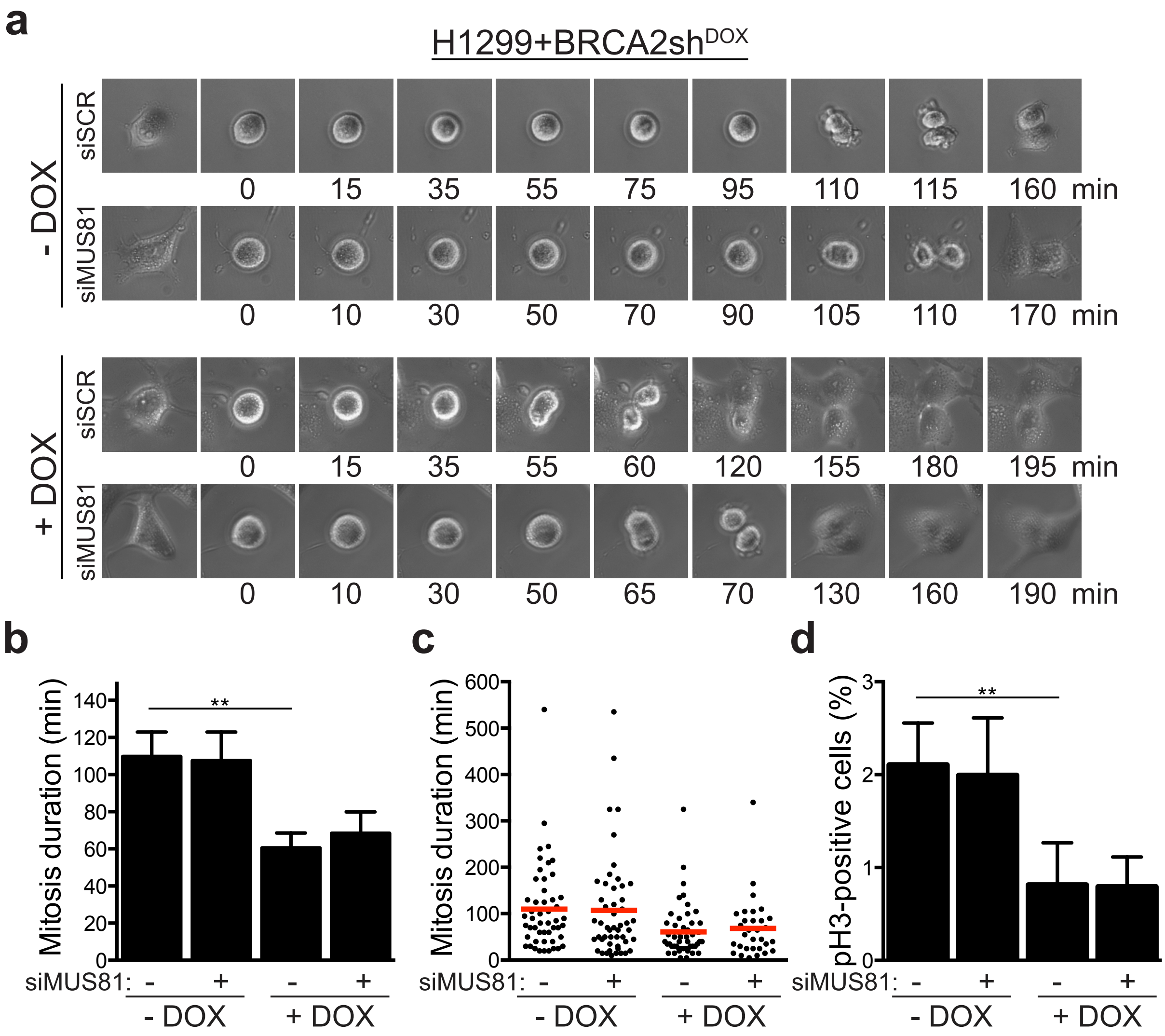

Supplementary Figure 6. Loss of BRCA2 reduces mitosis duration. (a) H1299 cells expressing a DOX-inducible BRCA2 shRNA were transfected with control or MUS81 siRNAs and examined by time-lapse microscopy after 7 days. Images were taken at 5-min intervals for 48 h. Representative images of progressive mitotic stages of indicated cells are shown. (b) Mean mitotic duration of cells treated as in (a). At least 30 cells were analysed for each condition. Error bars indicate SEM. **, $p < 0.01$ (unpaired two-tailed *t* test). (c) Mitotic duration of individual cells treated as in (a). Red bars indicate mean. (d) Cells treated as in (a) were processed after 24 h for histone H3 phospho-Ser10 staining. Error bars indicate SD (n=4). **, $p < 0.01$ (unpaired two-tailed *t* test).

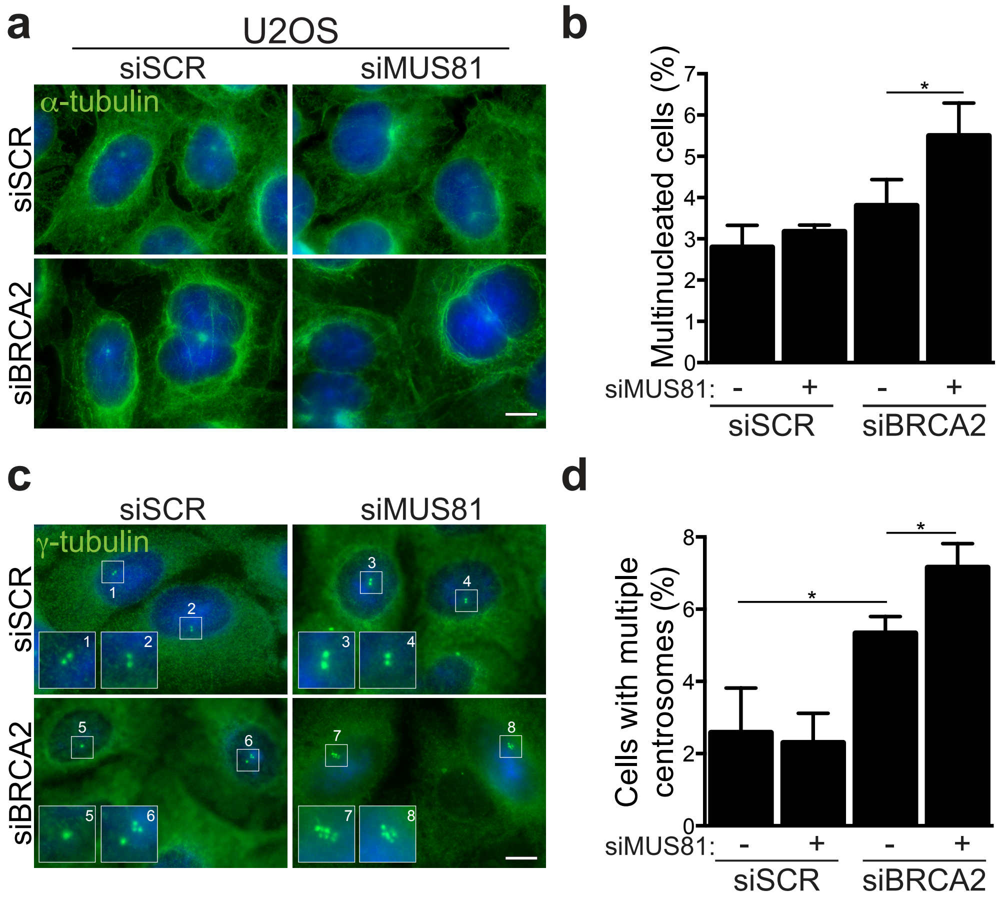

Supplementary Figure 7. MUS81 depletion in BRCA2-deficient U2OS cells results in cytokinesis failure. (a) U2OS cells were transfected with control, BRCA2 and/or MUS81 siRNAs and processed for immunostaining with α-tubulin after 72 h. Scale bar, 10 μm. (b) Quantification of multinucleated cells treated as in (a). Error bars indicate SD (n=3). *, $p < 0.05$ (unpaired two-tailed t test). (c) Cells treated as in (a) were processed for immunostaining with γ-tubulin after 72 h. Scale bar, 10 μm. (d) Quantification of cells with multiple centrosomes treated as in (c). Error bars indicate SD (n=2). *, $p < 0.05$ (unpaired two-tailed t test).

Figure 1b

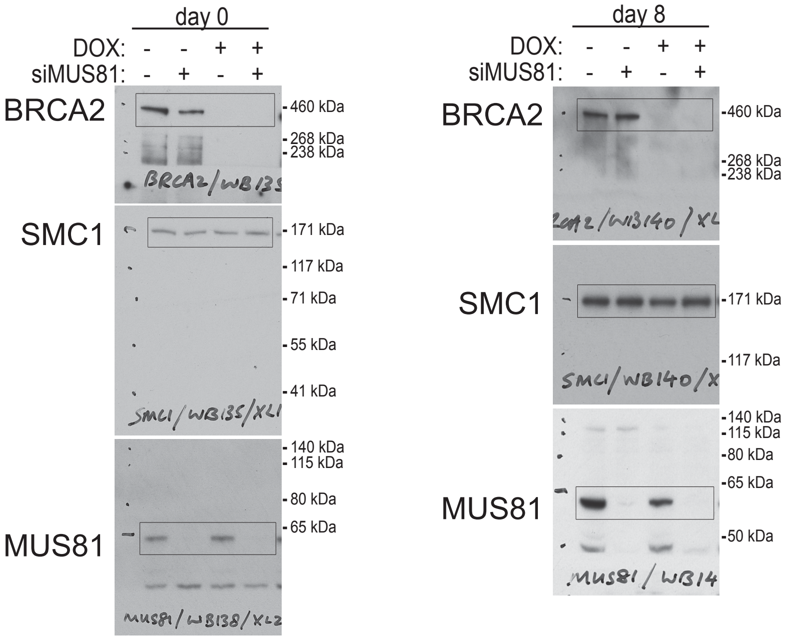

Figure 1c

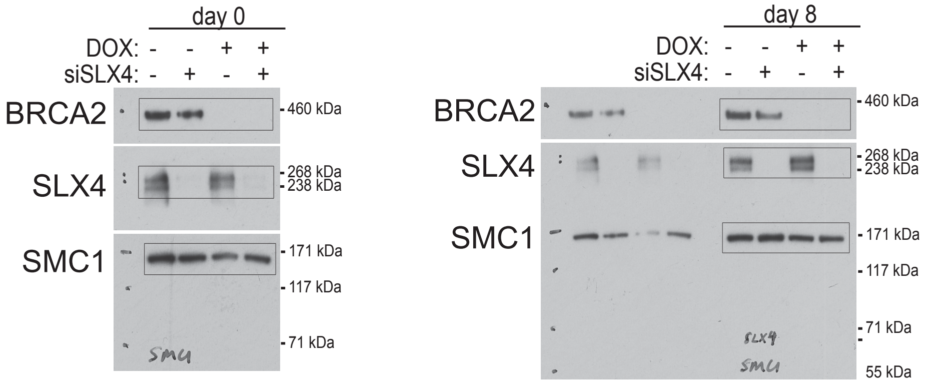

Figure 2c

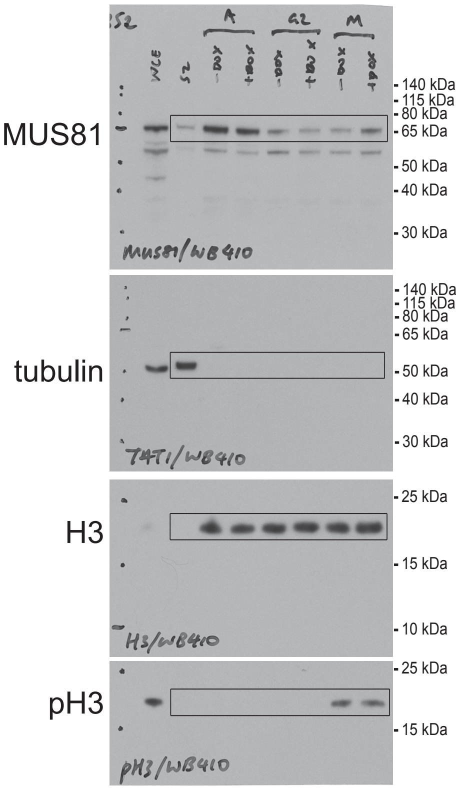

Figure 2e

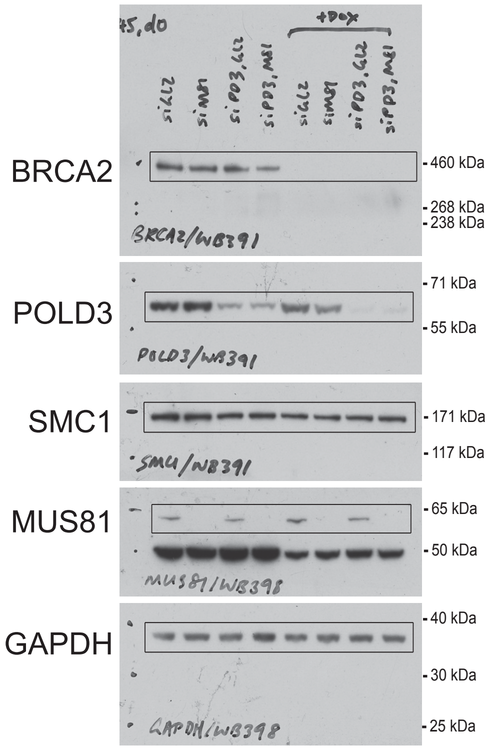

Supplementary Figure 8. Uncropped blots for Figures 1 and 2.

			Total number of fibers quantified	Mean fiber track length (µm)		
				Experiment		
				1	2	3
	- DOX	siSCR	524	12.72	14.56	15.72
		siMUS81	558	12.56	13.32	16.63
	+ DOX	siSCR	580	8.42	12.14	13.50
		siMUS81	542	6.72	9.76	8.31
+ WT	- DOX	siSCR	587	12.34	14.55	14.14
		siMUS81	543	11.99	13.45	14.71
	+ DOX	siSCR	550	9.15	9.94	12.44
		siMUS81	589	9.80	10.02	12.73
+ CI	- DOX	siSCR	544	13.15	14.37	14.22
		siMUS81	565	12.64	13.74	14.26
	+ DOX	siSCR	552	10.95	12.45	10.82
		siMUS81	600	8.50	10.39	9.05

Supplementary Table 1. Total number of fibers and mean fiber track length (µm) quantified in Fig. 1a.

		Total number of fibers quantified	Mean fiber track length (µm)		
			Experiment		
			1	2	3
- siBRCA2	siSCR	454	23.22	25.81	27.30
	siMUS81	309	25.82	27.96	26.16
+ siBRCA2	siSCR	319	27.51	27.13	22.74
	siMUS81	421	18.14	19.36	18.65

Supplementary Table 2. Total number of fibers and mean fiber track length (µm) quantified in Supplementary Fig. 1b.
